# Transcriptome Sequencing and Differential Gene Expression Analysis of Delayed Gland Morphogenesis in *Gossypium* *australe* during Seed Germination

**DOI:** 10.1371/journal.pone.0075323

**Published:** 2013-09-20

**Authors:** Tao Tao, Liang Zhao, Yuanda Lv, Jiedan Chen, Yan Hu, Tianzhen Zhang, Baoliang Zhou

**Affiliations:** State Key Laboratory of Crop Genetics & Germplasm Enhancement, MOE Hybrid Cotton R&D Engineering Research Center, Nanjing Agricultural University, Nanjing, Jiangsu, People’s Republic of China; New Mexico State University, United States of America

## Abstract

The genus *Gossypium* is a globally important crop that is used to produce textiles, oil and protein. However, gossypol, which is found in cultivated cottonseed, is toxic to humans and non-ruminant animals. Efforts have been made to breed improved cultivated cotton with lower gossypol content. The delayed gland morphogenesis trait possessed by some Australian wild cotton species may enable the widespread, direct usage of cottonseed. However, the mechanisms about the delayed gland morphogenesis are still unknown. Here, we sequenced the first Australian wild cotton species ( *Gossypium* *australe* ) and a diploid cotton species ( *Gossypium* *arboreum* ) using the Illumina Hiseq 2000 RNA-seq platform to help elucidate the mechanisms underlying gossypol synthesis and gland development. Paired-end Illumina short reads were *de novo* assembled into 226,184, 213,257 and 275,434 transcripts, clustering into 61,048, 47,908 and 72,985 individual clusters with N50 lengths of 1,710 bp, 1544 BP and 1,743 bp, respectively. The clustered *Unigenes* were searched against three public protein databases (TrEMBL, SwissProt and RefSeq) and the nucleotide and protein sequences of *Gossypium* *raimondii* using BLASTx and BLASTn. A total of 21,987, 17,209 and 25,325 *Unigenes* were annotated. Of these, 18,766 (85.4%), 14,552 (84.6%) and 21,374 (84.4%) *Unigenes* could be assigned to GO-term classifications. We identified and analyzed 13,884 differentially expressed *Unigenes* by clustering and functional enrichment. Terpenoid-related biosynthesis pathways showed differentially regulated expression patterns between the two cotton species. Phylogenetic analysis of the terpene synthases family was also carried out to clarify the classifications of TPSs. RNA-seq data from two distinct cotton species provide comprehensive transcriptome annotation resources and global gene expression profiles during seed germination and gland and gossypol formation. These data may be used to further elucidate various mechanisms and help promote the usage of cottonseed.

## Introduction

Cotton is a globally appreciated, remarkable economic crop, as cotton produces a natural textile fiber. In addition, worldwide cottonseed production has the potential to provide protein for half a billion people annually if cottonseed could be directly consumed as a food. However, the presence of gossypol within the pigment glands of cultivated cotton limits the usage of cottonseed due to its toxicity to humans and non-ruminant animals. Gossypol is a yellowish phenolic compound that occurs naturally in certain species of cotton plants of the family Malvaceae and contributes to the self-defense mechanisms of cotton, protecting the plant from pests, diseases and abiotic stresses [1,2]. Gossypol is synthesized in cotton roots and transported and stored within pigment glands of cotton above ground [3]. This important compound also has antitumor activity and possess contraceptive properties, which makes it unique and commercially valuable [4,5].

Many efforts have been made by geneticists and breeders to eliminate gossypol within cottonseeds. However, gossypol content is highly related to insect resistance. The glanded and glandless cotton species exhibit great differences in the amount of insect feeding [6]. Thus, breeding a high-yielding “glandless-seed” and “glanded-plant” cultivar has become an area of interest for researchers. Interestingly, some wild diploid cotton species in Australia, such as *G* *. australe* , *G* *. bickii* and *G* *. sturtianum* , possess a unique characteristic, namely, that the pigment glands only appear after seed germination; thus, the dormant seeds of these species lack gossypol [7]. This distinguishing characteristic, known as the delayed gland morphogenesis trait, has the potential to enable the large-scale, direct usage of cottonseed. Various efforts have been made to introduce this unique characteristic of wild Australian cotton species into cultivated tetraploid cotton [8,9], but the cultivars with the delayed gland morphogenesis trait have not been developed by now.

Inheritance studies have been carried out to elucidate the genetics of cotton gland and gossypol formation. Various results were obtained from these studies due to the differences between diverse cotton species. Previous studies have shown that in lines of Hopi cotton, the glandless trait is controlled by recessive genes, *gl*_2_ and *gl*_3_ [10,11]. However, in Hai 1, the dominant gene *GL*_*2*_^*e*^ is mainly responsible for this trait [12,13]. The diversity of glandless trait inheritance indicates the complexity of gland formation and regulation across different cotton species. Further studies are needed to better understand the mechanisms underlying gland development.

Terpenes comprise the largest class of natural products and participate mainly in secondary or primary metabolism in processes such as sterol and carotene biosynthesis. Plants accumulate terpenes, some of which are released for various purposes such as plant defense against herbivores, to attract pollinators and in response to stress [14]. Sesquiterpenoids are the most commonly found terpenes that accumulate within pigment glands of cotton species, including gossypol, and can be classified as phytoalexins due to their potential role in plant resistance [15,16]. Isopentenyl diphosphate (IPP) is the common precursor of all terpenes and is synthesized in plastids via the cytosol-localized mevalonic (MEV) pathway and the MEP/DOXP pathway. Geranyl diphosphate (GPP), farnesyl diphosphate (FPP) and geranylgeranyl diphosphate (GGPP) are the precursors of monoterpenes, sesquiterpenes and diterpenes, respectively [17].

Various genes associated with gossypol and glands within the terpenoid biosynthesis pathways have been cloned. The cadinene enzyme was first purified from a glandless cotton mutant by Davis et al. as a soluble hydrophobic monomer with a molecular mass of 64 to 65 kD [18]. Chen et al. first cloned and functionally characterized a (+)-δ-cadinene synthase (CDN1-XC14/U23205) from the A-genome diploid cotton *G* *. arboreum* . Two major subfamilies of the *Gossypium* cadinene synthase multigene family, namely CDN1-A and CDN1-C, were proposed according to sequence similarities [19-25]. The next step in gossypol biosynthesis involves hydroxylation of (+)-δ-cadinene to 8-hydroxy-(+)-δ-cadinene, which is catalyzed by (+)-δ-cadinene-8-hydroxylase, a cytochrome P450 mono-oxygenase (CYP706B1); the gene encoding this enzyme was cloned and characterized by Luo et al. [26]. Some transcription factors are important regulatory molecules involved in gland and gossypol formation, such as GaWRKY1 [27], MYC2 [28], RanBP2 zinc finger protein [29] and others, indicating that active binding events occur during gland and gossypol development.

Next-generation sequencing (NGS) technology has recently been widely employed in diverse studies to provide a comprehensive overview of the genomes and transcriptomes of certain species. Since NGS technology has the advantage of producing massive amounts of data at a low cost, deep-sequencing technology is currently undergoing rapid development. Three sequencing platforms have been employed in the majority of sequencing projects, namely Roche 454, Illumina Hiseq/Miseq and ABI SOLiD. Since the development of NGS technology, many genomes have been sequenced, including plants such as grapevine [30], tomato [31], potato [32] and others. Such studies provide large quantities of valuable information to help further elucidate complex mechanisms that occur within certain species. RNA-seq is a revolutionary tool for transcriptome profiling that uses deep-sequencing technologies. RNA-seq can be used for various purposes, such as transcript quantification, comprehensive annotation of transcriptomes, reannotation of genomes, identification of novel transcripts and alternative splicing events [33-35] and detection of polymorphisms at the transcriptome level [36,37]. RNA-seq can be performed with or without a reference genome, which makes this technique a perfect alternative for analyzing non-model species that lack fully described genomic sequences.

Recently, the genome sequence of *G* *. raimondii* (2n = 2x = D_5_D_5_ = 26), which is believed to be one of the ancestors of currently cultivated allotetraploid cotton, has been accomplished [38,39], providing cotton geneticists worldwide with a valuable resource to better explore the biological networks of this important crop. In this study, we analyzed the first wild Australian cotton species ( *G* *. australe* ), which possesses the unique delayed gland morphogenesis trait as well as *Verticillium* wilt disease- and stress-resistance characteristics [40], along with an A-genome diploid cotton species ( *G* *. arboreum* ), using the Illumina Hiseq 2000 RNA-seq platform. The paired-end (PE) reads were used for *de novo* assembly due to the differences between the three chromosome sets (A, D, G). The objective of this study was to perform a comprehensive comparison of two highly diverse cotton species during seed germination and to identify transcripts that may be important for gland and gossypol formation. The results of this study may be useful for further elucidating seed developmental mechanism, as well as the formation of glands and gossypol, at the whole-transcriptome level.

## Materials and Methods

### Plant Material and RNA Extraction

Plants of diploid cotton ( *Gossypium* *arboreum* L. cv. Jianglinzhongmian and *G* *. australe* F. Muell) were grown in a greenhouse at Nanjing Agricultural University, China. Delinting treatment was applied to mature seeds using H_2_SO_4_ at a concentration of 80%. The sundried seeds were then sterilized with 70% ethanol for 30s and 30% H_2_O_2_ for 1 h, followed by washing with sterile water. The seed coats were removed from the sterilized seeds by soaking the seeds in sterile water for 18 h, followed by germination in the dark at 28°C. After germination for 5 h, 15 h or 30 h, samples of *G* *. arboreum* L. (2n = 2x = A_2_A_2_ = 26) and *G* *. australe* F. Muell (2n = 2x = G_2_G_2_ = 26) were immediately frozen and stored at -70°C.

Total RNA was extracted from these six samples according to the modified CTAB-sour phenol extraction method [41]. Each RNA sample was treated with RNase-free DNase I (Takara Bio, Dalian, China) after extraction to remove residual DNA. The RNA quality and purity were assessed according to the OD_260/230_ ratio and the RNA integrity number (RIN) using a Qubit^®^ 2.0 Fluorometer (Invitrogen, Carlsbad, CA, U.S.) and an Agilent 2100 Bioanalyzer (Agilent Technologies, U.S.).

### cDNA Library Preparation for Illumina Sequencing

The cDNA libraries of the six high-quality RNA samples (RIN > 8) were prepared following the manufacturer’s instructions in the Illumina^®^ TruSeq^TM^ RNA Sample Preparation Kit (Illumina Inc. San Diego, CA, U.S.) using the Low-Throughput Protocol. Poly-T oligo-attached magnetic beads were used to purify the poly-A-containing mRNA molecules. The mRNA was fragmented into 200–500 bp pieces using divalent cations at an elevated temperature (94°C for 6 min). The cleaved RNA fragments were copied into first-strand cDNA using SuperScript II Reverse Transcriptase (Life Technology Inc., CA, U.S.) and random hexamer-primers with the following program: 25°C for 10 min, 42°C for 50 min and 70°C for 15 min. Second-strand cDNA was synthesized using DNA polymerase I and RNase H. These cDNA fragments were then end-repaired with the addition of a single ‘A’ base, followed by ligation of the adapters. The products were then purified following the instructions in the MinElute PCR Purification Kit (Qiagen, Düsseldorf, Germany) and eluted in 10 µL of Qiagen EB buffer. The eluted fragments were assessed by size on a 2% agarose gel to select fragments in the range of 400 bp ± 50 bp and retrieved using a MinElute Gel Extraction Kit (Qiagen, Düsseldorf, Germany). PCR of the selected fragments was performed using PCR Master Mix and Primer Cocktail in a Sample Preparation Kit (Illumina Inc.) using the following program: 98°C for 30 s; 15 cycles of 98°C for 10 s, 60°C for 30 s, 72°C for 30 s; 72°C for 5 min; hold at 4°C. The PCR products were purified using a MinElute PCR Purification Kit (Qiagen) in a final sample volume of 30 µL. The tagged cDNA libraries were loaded onto flow cell channels at a concentration of 2–4 pM and used for 2 × 100-bp paired-end sequencing on a single lane of the Illumina HiSeq 2000 Sequencing Platform (Illumina Inc., CA, U.S.). The samples were demultiplexed, and the indexed adapter paired reads were trimmed using CASAVA v1.8.2 software (Illumina Inc.).

### Data Preprocessing and *De novo* Transcriptome Assembly

The raw FASTQ format data sets generated from CASAVA v1.8.2 were first assessed for quality using FASTQC v0.10.1 [42] and FASTX toolkit v0.0.13 [43]. Reads contaminated with Illumina adapters were detected and removed using Trimmomatic software (Released Version 0.22, http://www.usadellab.org/cms/index/php?page=trimmomatic). Poor-quality reads (Phred score < 20) were trimmed from both ends with SolexaQA packages v2.0 [44]; only the reads with lengths ≥ 25 bp on both sides of the paired-end format were subjected to further analysis. All sequencing data have been deposited in SRA (www.ncbi.nlm.nih.gov/sra). The accession number is SRR927415.The remaining quality paired-end reads were *de novo* assembled into transcripts with the Trinity program (Released on 2012-10-05) [45]. In-house Perl scripts were written to extract the longest transcript in each cluster as a unigene for downstream analysis. Representative extracted transcripts were then searched against human, bacterial and rRNA sequence contamination using the web-based version of DeconSeq (http://edwards.sdsu.edu/cgi-bin/deconseq/deconseq.cgi) [46] with default parameters.

### Function Annotation and Classification of Assembled Transcriptomes

Annotations of the distinct *Unigenes* were performed using the BLASTx search program in the stand-alone NCBI-BLAST package v2.2.26+ [47]. The assembled contig sets were compared against Uniprot/Swissprot (released on 11-2012), Uniprot/TrEMBL (released on 11-2012) [48] and RefSeq-Plant (released on 11-2012 with plant data sets only) [49] protein databases with an expect E-value cutoff ≤ 1e-6. *Unigenes* were also searched against the recently published CDS and protein sequences within the *G* *. raimondii* genome project hosted by JGI (ftp://ftp.jgi-psf.org/pub/compgen/phytozome/v9.0/Graimondii) using BLASTx and BLASTn.

The BLASTx results were then combined and imported into Blast2GO software v2.6.2 [50] for gene ontology (GO) term analysis, describing biological process, molecular function and cellular component. The top 20 Blast hits with a cutoff E-value of 1e-6 and similarity cut-off of 55% were determined for GO annotation. The obtained annotations were enriched and refined using ANNEX; level 2 of the GO annotations are presented. GO-slim terms analysis was also performed using Blast2GO to obtain a broad overview of the ontology distributions. The Plant-slims developed by the *Arabidopsis* Information Resource was specifically chosen to implement the GO-slim step.

Moreover, the enzyme commission numbers (EC) of the corresponding GO annotated sequences were also obtained with an E-value cutoff of 1e-6. KEGG pathways were assigned to the assembled *Unigenes* using the online KEGG Automatic Annotation Server (KAAS, http://www.genome.jp/tools/kaas) [51]. The KEGG Ortholog assignments and pathway maps were obtained using the bidirectional best hit method (BBH) on the KAAS website.

### Transcriptome Quantification

In many comparative analysis pipelines, including variant calling, isoform quantitation and differential gene expression, the first committed step is aligning the reads back to a reference genome or transcriptome. Here, a newly modified Burrows-Wheeler transform (BWT) aligner, Bowtie2 v 2.0.1 [52], was applied for this purpose. The quality trimmed paired-end reads were aligned back to the assembled transcriptome with Bowtie2 and alignment results were converted to BAM format using SAMtools [53].

Normalizing and quantifying gene expression levels from ambiguous alignment results are statistical challenges when performing high-throughput RNA sequencing. The recently developed software package “eXpress” [54] v1.2.1 was used to accurately quantify the abundance of transcript-level sequences and to calculate the FPKM (fragments per kilobase of transcript per million mapped reads); only transcripts with an FPKM ≥ 1 were considered to be expressed.

### Differential Gene Expression Analysis

Differentially expressed genes were called via edgeR package v3.0.8 [55]. The raw counts generated from the eXpress program were imported into edgeR to determine the significance of transcript-level expression. False discovery rate (FDR) was used to determine the threshold of the *P*-value in multiple tests. FDR ≤ 0.001 and the absolute value of |log2Ratio| ≥ 1 were considered to be the cutoff threshold to determine the significance of expression. GO enrichment analysis of differentially expressed genes was performed using Blast2GO software. A *P*-value cutoff value of 0.05 during the Fisher’s exact test was used for GO enrichments against the annotated *Unigenes* .

Significantly regulated genes were also assessed by applying the Clustering algorithm. Hierarchical clustering was performed using Cluster v3.0 software [56]. Gene expression values were extracted from the edgeR-normalized FPKM data sets. Matrix distance for expression heatmap was calculated with Euclidean distance and complete-linkage methods after original FPKM values were log-transformed and centered. A heatmap was constructed using TreeView v1.1.6 [57] and MeV v4.8.1 [58]. The expression patterns of the self-defined clusters were plotted with R (2.15) scripts.

### Phylogenetic Analysis of Terpene Synthase Genes

Terpene synthase-related genes of assembled “A” and “G” transcriptomes were predicted using the *getorf* program in the EMBOSS software package [59]. TPSs of other plant species were downloaded from NCBI. The *G* *. raimondii* protein data set was used to obtain the TPS protein sequences of the D genome. The hmmsearch program in HMMER3.0 [60] was used to search for amino acid sequences that contain the Pfam Terpene Synthase domains PF03936 and PF01397 [61]. MUSLE was used for multiple sequence alignments, and a Maximum Likelihood Tree was drawn with MEGA v5.1 [62]. The Java program FigTree v1.4.0 (http://tree.bio.ed.ac.uk/software/figtree/) was used to modify and generate the final phylogenetic tree.

### Real-time Quantitative (qRT-PCR) Validation

The RNA sequencing samples that were isolated were also used to perform real-time quantitative (qRT-PCR) analysis. First-strand cDNA was synthesized using M-MLV Reverse Transcriptase (Promega, U.S.). Gene-specific primers were designed according to the comparison of the three assembled unigene sequences using Jalview [63], and Primer Premier 5.0 (Premier Biosoft International, Palo Alto, CA, U.S.) was applied to determine the primer sequences. *Histone3* (AF024716) was used as an internal control. The qRT-PCR was carried out using iQ SYBR Green Supremix (Bio-Rad, USA) according to the manufacturer’s instructions. The thermal cycle conditions for PCR were as follows: 94°C for 3 min, 30 cycles including 94°C for 15 sec, 60°C for 30 sec and 72°C for 30 sec. The relative expression levels were calculated using the 2^-ΔΔCt^ method [64].

## Results and Discussion

### Illumina Sequencing and *De novo* Assembly

**Figure 1:**
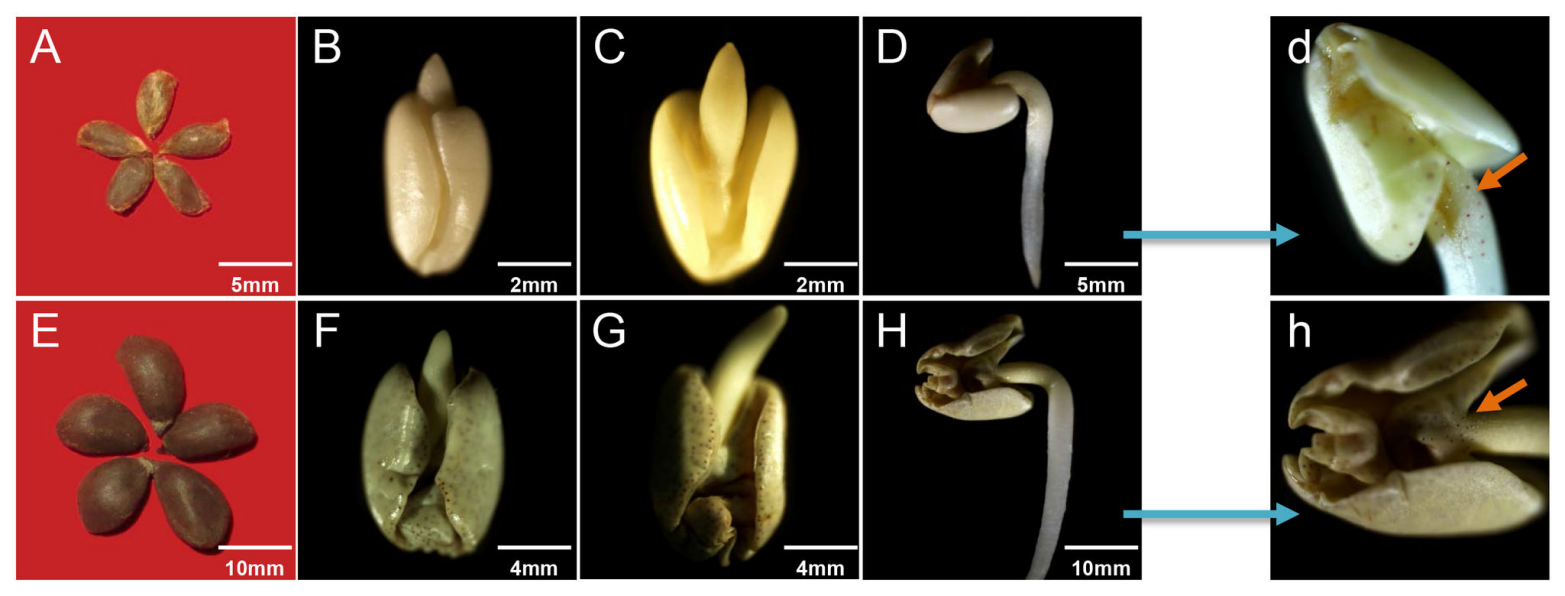
Stereo microscope scans of different seed germination stages. (A) and (E) are the delinted seeds of *Gossypium* *australe* and *Gossypium* *arboreum* , respectively. (B) (C) and (D) are the three germination stages of *G* *. australe* , i.e., 5 h, 15 h and 30 h. (F), (G) and (H) are the same three germination stages of *G* *. arboreum* . (d) and (h) are magnified images of (D) and (H).

Mature seeds of *Gossypium* *arboreum* L. (2n = 2x = A_2_A_2_ = 26) and *Gossypium* *australe* F. Muell (2n = 2x = G_2_G_2_ = 26) were first delinted using highly concentrated H_2_SO_4_ and treated with ethanol and H_2_O_2_ to break dormancy. Preliminary tests were carried out, and three targeted germination stages (i.e., 5 h-G1/A1, 15 h-G2/A2 and 30 h-G3/A3) were subjected to RNA sequencing (See Material and Methods) using the Illumina HiSeq 2000 Platform. Morphological characteristics of seed germination were observed using a stereo microscope (Figure **1**). The pigment glands of *G* *. australe* could be observed after the seeds were germinated for more than 24 h, which is consistent with the results of previous studies [65].

Three cDNA libraries of each cotton species were bar code tagged and sequenced on one lane of the flow cell. A total of 142,880,698 (**G1 & G2 & G3**) and 252,661,798 (**A1 & A2 & A3**) raw paired-end reads with a length of 101 bp, corresponding to *G* *. australe* and *G* *. arboreum* , respectively, were generated, resulting in 35 GB and 62 GB, respectively. The raw reads were then trimmed with Illumina adapters using various techniques, and low quality bases were filtered out. The statistics of both raw and trimmed sequencing data are summarized in Table **1**. The manually selected insert library size was approximately 380 bp.

**Table 1:** Statistics of transcriptome sequencing.

**Library**	**Insert size (bp**)	**Raw nt (Gb**)	**Raw read pairs**	**Trimmed nt (Gb**)	**Trimmed read pairs** **(both ends≥ 25bp**)
**G1**	380	6.80	13,883,940	3.03	7,292,030
**G2**	380	17.32	35,346,542	8.61	20,654,711
**G3**	380	10.88	22,209,867	5.49	13,108,013
**A1**	380	14.90	30,426,341	7.42	17,863,832
**A2**	380	32.26	65,824,214	16.36	39,236,996
**A3**	380	14.74	30,080,344	7.48	17,819,814

**Figure 2:**
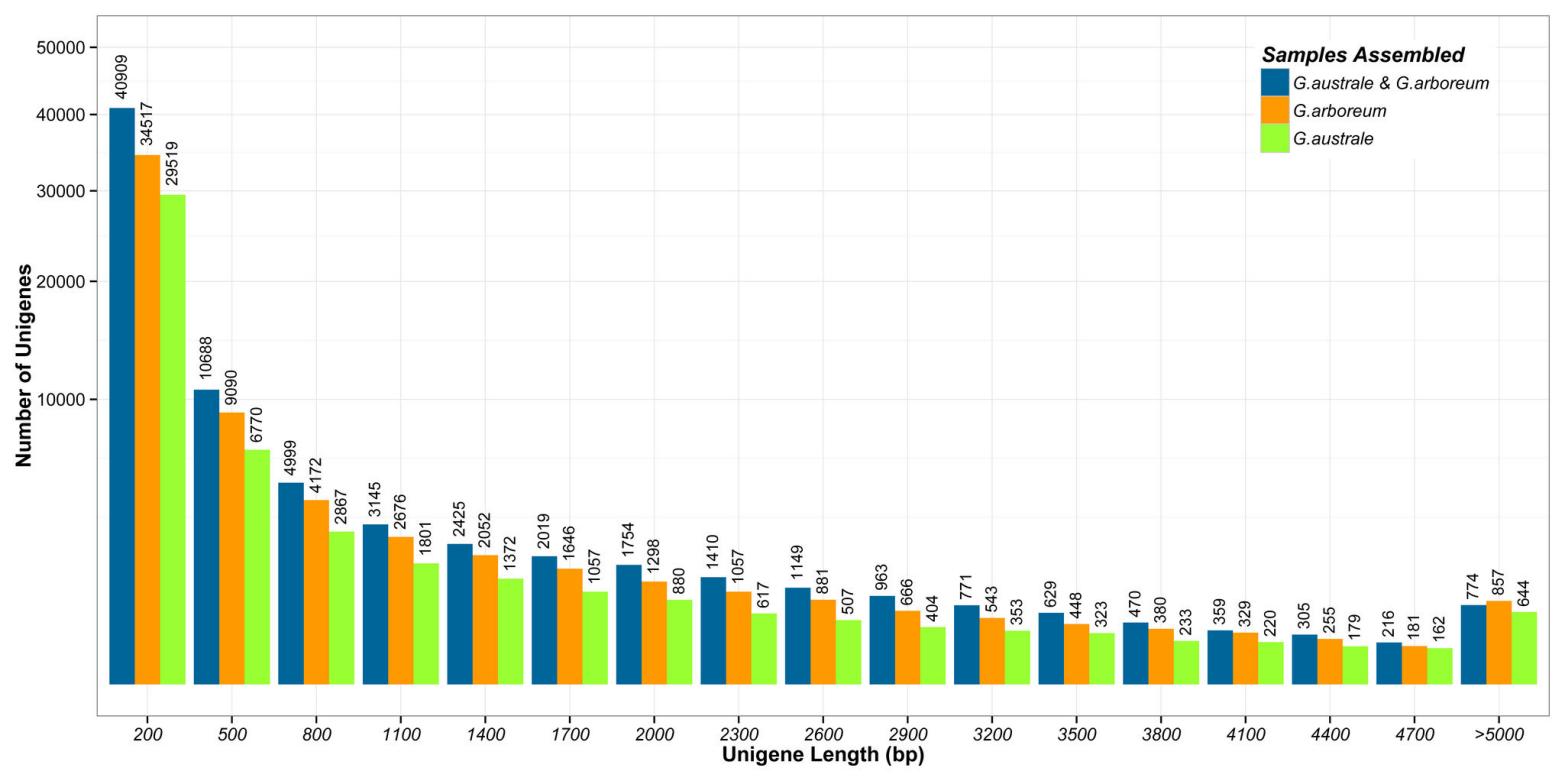
Length distribution of Trinity assembly for *Unigenes* of individual and combined data sets. Six data sets generated from three different seed germination stages of *G*. *australe* and *G*. *arboreum* were assembled using Trinity, length distribution of Trinity assembly from 200bp to >5000bp were presented (three data sets of *G* *. australe* were merged for assembly (green), as well as those of *G* *. arboreum* (orange), and six data sets were also assembled together (blue).

The quality trimmed reads (Q ≥ 20) were then *de novo* assembled into transcripts using Trinity, with a fixed k-mer of 25. We applied the “Reduce” option within the recently modified version of the Trinity software package to reduce redundancy in assembled transcriptomes. The cDNA libraries of three different stages of germination were pooled together for Trinity assembler to represent the whole transcriptome during germination for both *G* *. arboreum* and *G* *. australe* . With the purpose of detecting differentially expressed genes between *G* *. arboreum* and *G* *. australe* during germination, the six cDNA libraries were also assembled together as a reference transcriptome using Trinity. The three data sets, corresponding to *G* *. arboreum* , *G* *. australe* and *G* *. australe* and *G* *. arboreum* , were assembled into 226,184, 213,257 and 275,434 transcripts, respectively, clustering into 61,048, 47,908 and 72,985 individual clusters (Table **2**). The transcriptome assembly results may be redundant due to various alternative splicing events as well as misassemblies [66-69]. Therefore, we manually selected the longest transcript in each cluster as the representative based on custom Perl scripts, hoping to obtain a broad view of the three assembled transcriptomes while at the same time simplifying the data sets. The *Unigenes* were evaluated for GC content, N50 and contig length distribution based on the in-house Perl script (Table **2**). The GC contents of the three unigene sets were all approximately 37%–38%, which is considered to be normal, as cotton possesses a relatively low GC content [70-72]. The N50s of the *Unigenes* were remarkably high, achieving 1,710, 1,544 and 1,743, respectively, which may be due to the high sequencing depth. A fairly large number (34,517 of 61,084, 29,519 of 47,908 and 40,909 of 72,985) of assembled *Unigenes* were between 200 bp and 500 bp in length, indicating the presence of assembled fragments (Figure **2**).

**Table 2:** Summary of *de novo* assembly.

**Transcriptome assembled**	**Transcripts ≥ 200bp**	**Transcripts ≥ 500bp**	**No. of *Unigenes* **	**N50 of *Unigenes* **	**GC% of *Unigenes***
** *G* *. arboreum* **	226,184	157,539	61,048	1,710	37.67
** *G* *. australe* **	213,257	147,219	47,908	1,544	37.81
** *G* *. australe* *&* *G* *. arboreum* **	275,434	177,118	72,985	1,743	37.39

Any rRNA sequences, as well as bacterial and human transcriptome contamination, were scanned using the web-based software DeconSeq with default parameters. A total of 13 (0.02%), 22 (0.05%) and 37 (0.05%) *Unigenes* were identified as contamination, corresponding to the three unigene sets. However, the *Unigenes* confirmed by DeconSeq were short and were likely to be assembled fragments.

### Functional Annotation and Classification

**Figure 3:**
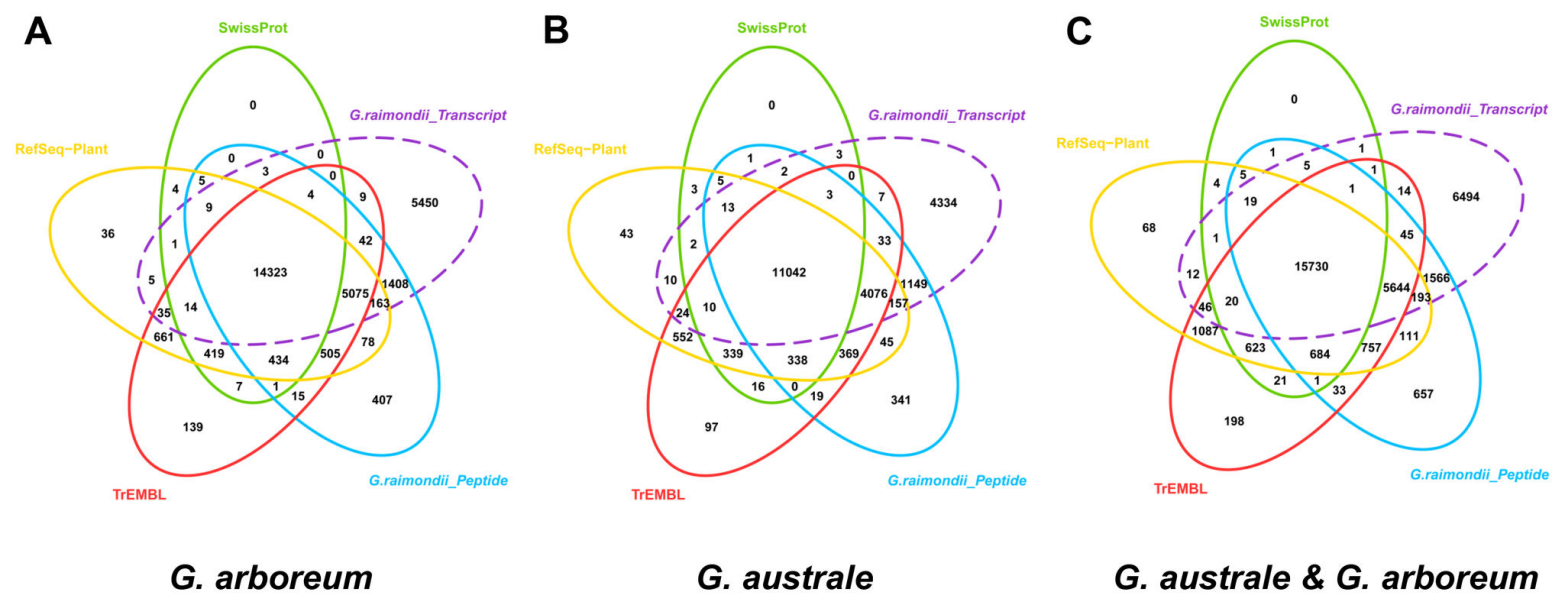
BLASTx and BLASTn annotation against various databases. Protein databases including Swissprot (green), TrEMBL (red) and RefSeq-Plant (yellow) were used for BLASTx annotation. The peptide (blue) and transcript (purple) sequences of *G* *. raimondii* (JGI) were applied for both BLASTx and BLASTn with E-value ≤ 1e-6. A, B and C represent the annotation results of *G* *. arboreum* , *G* *. australe* , *G* *. arboreum* & *G* *. australe* assemblies, respectively. The number of common annotated genes is shown in the overlapping segment of the venn diagrams.

We applied various approaches to validate the assemblies in order to obtain comprehensive descriptions of the assembled transcriptomes. The three assembled unigene sets (designated “A” for *G* *. arboreum* , “G” for *G* *. austral* and “A & G” for *G* *. arboreum* and *G* *. australe* for simplification, according to the genome type) were first used for homology searching against the Uniprot/Swissprot, Uniprot/TrEMBL and NCBI RefSeq Plant protein databases using the BLASTx algorithm, with an E-value cutoff of 1e-06. More than 94% of the annotated *Unigenes* in all three sets had E-values < 1e-10, indicating the reliability of the annotated results. We combined the annotation results from all three protein databases and obtained 21,987 (“A”), 17,209 (“G”) and 25,325 (“A & G”) *Unigenes* with BLASTx hits. The *Unigenes* were also searched against the CDS and protein sequences within the *G* *. raimondii* genome project using BLASTx and BLASTn, with an E-value cutoff of 1e-6 and 1e-10, respectively. More *Unigenes* were annotated due to sequence similarities (Figure **3**), indicating unique cotton gene models. The results show that applying diverse databases can enrich annotations, and certain species may have unique gene models that can only be annotated with closer relatives. There were also a fairly large number of sequences (39,036 of 61,084, 30,699 of 47,908, and 47,660 of 72,985) that were not annotated to any of the databases mentioned. These sequences may represent transcript fragments that were assembled that did not represent full-length domains, as well as noncoding RNAs or misassemblies [35,68,69].

**Figure 4:**
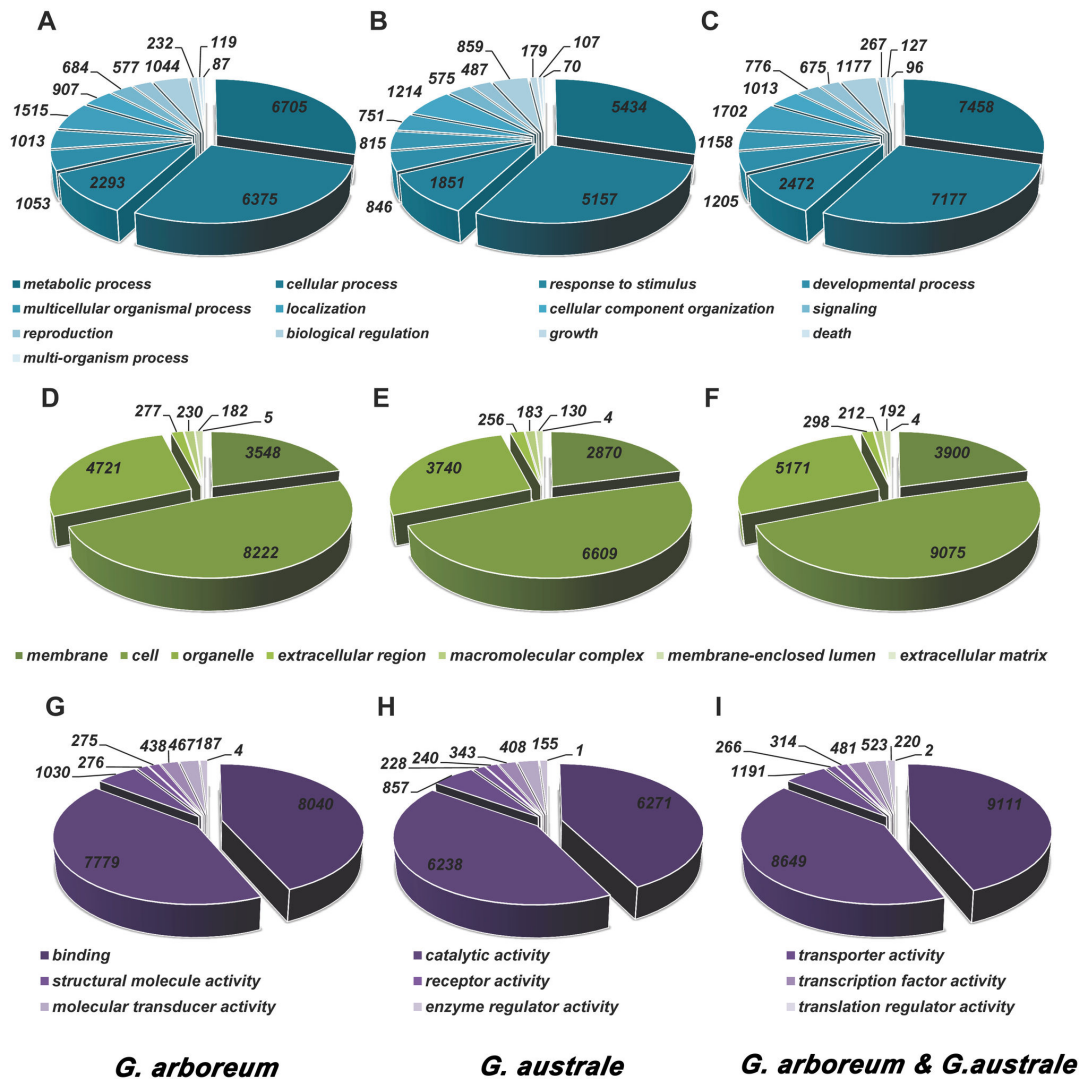
Distribution of GO-slim functional classification. (A) (D) and (G) represent the GO-slim classification of *G* *. arboreum* ; (B), (E) and (H) represent *G* *. australe* and (C), (F) and (I) represent the combined assembly ( *G* *. arboreum* & *G* *. australe* ). (A) (B) and (C) are the distribution of the level 2 Biological Process within GO-slim classification; (D), (E) and (F) are the level 2 Cellular Component distribution and (G), (H) and (I) are the level 2 Molecular Function distribution. The pie charts corresponding to the detailed GO-slim classification are arranged clockwise.

The annotated *Unigenes* were then assigned to Gene Othology (GO) terms for functional classification. Three main categories of GO classification, i.e., biological process, molecular function and cellular component, were analyzed separately to learn as much as possible about their functional distribution. A total of 18,766 (85.4%, “A”), 14,552 (84.6%, “G”) and 21,374 (84.4%, “A & G”) of the annotated *Unigenes* could be assigned to one or more GO term. To simplify the functional distribution, the annotated sequences were assigned to GO-slim terms [73] of plants to obtain a “thin” version of classification. Cellular process (GO:0009987) and metabolic process (GO:0008152) within biological process, binding activity (GO:0005488) and catalytic activity (GO:0003824) within molecular function and cells (GO:0005623) and organelles (GO:0043226) within cellular component were the most representative level 2 GO terms in all three data sets (Figure **4**). All annotated sequences were then associated with enzyme codes (ECs), which returned 1,202 (“A”), 1,121 (“G”) and 1,202 (“A & G”) unique EC numbers.

To further identify the biological pathways that are active during seed germination, unigene sets were searched against pathway collections in the Kyoto Encyclopedia of Genes and Genomes (KEGG) database. A total of 293 (“A”), 295 (“G”) and 296 (“A & G”) pathways were predicted using online annotation software. The most representative pathways included biosynthesis of secondary metabolites, involving terpene backbone biosynthesis and starch and sucrose metabolism. RNA transports, as well as spliceosome pathways, were also prominent within the mapping results.

### Comparative Analysis of Differential Expression during Germination

To better understand the dynamic performance between the two different transcriptomes during seed germination, abundance estimation was applied to quantify the expression levels of the six sequenced libraries. We first aligned the paired-end reads back to the combined assembled transcriptome (i.e., *G. arboreum* *&* *G* *. australe* ) using Bowtie2. The alignment results were retrieved and pooled into the newly published quantification software “eXpress”. Compared with previous quantification software such as RSEM and Cufflinks, “eXpress” excels in that it employs a sequence-bias model and specificity against *de novo* assembly workflow without the dependency of the genome background [54]. The FPKM (fragments per kilobase of transcript per million mapped reads) of each unigene were calculated and extracted from the estimation results.

**Figure 5:**
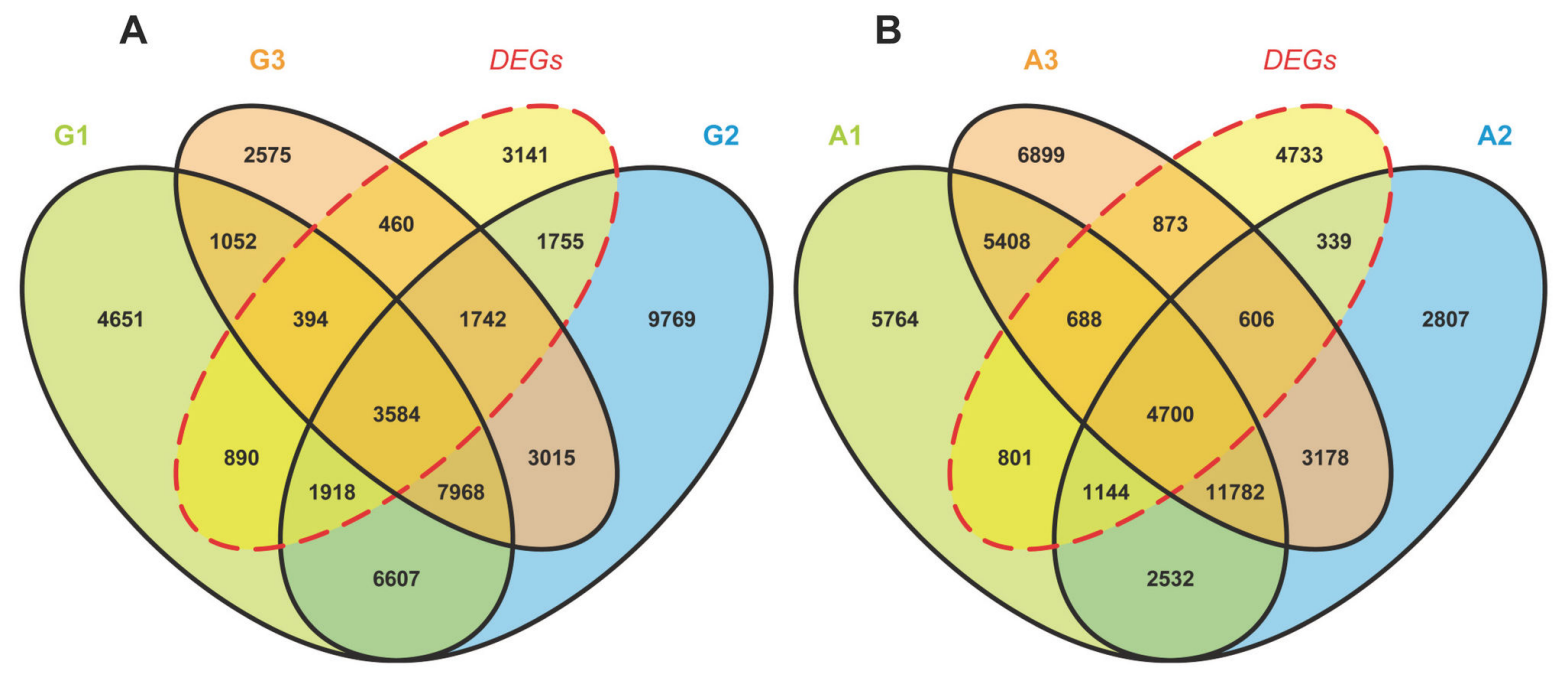
Venn diagrams of expressed genes and DEGs. (A) (B) are venn diagrams of *G* *. australe* and G. arboreum illustrating the relationship between total expressed *Unigenes* (FPKM ≥ 1) and DEGs detected by bowtie2-eXpress workflow. G1/A1, G2/A2, G3/A3 represent the number of expressed *Unigenes* in three germination stages. The individual and overlapping areas in venn diagrams represent the number of specifically expressed and co-expressed *Unigenes* between different stages.

**Figure 6:**
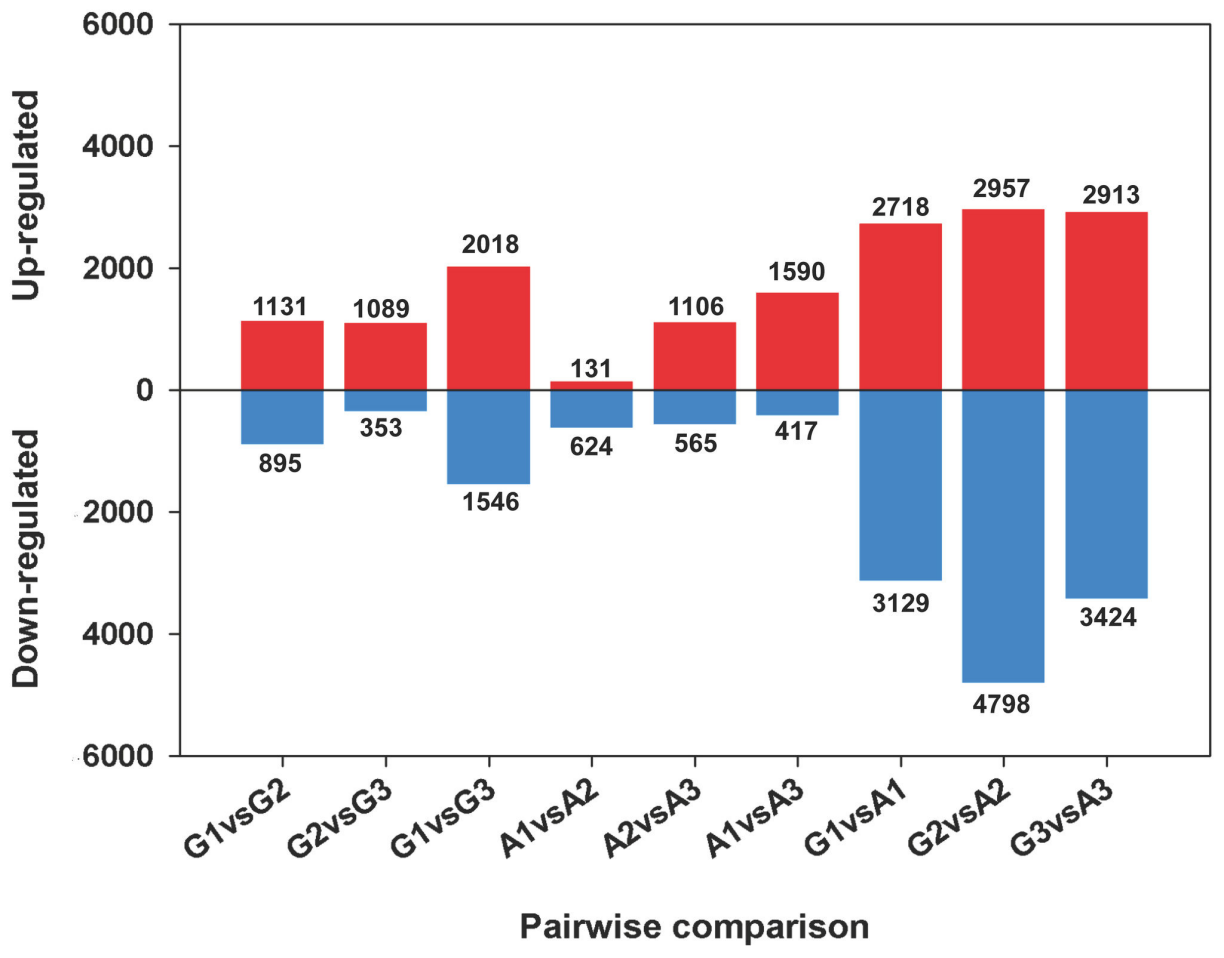
Bar graph of up- and downregulated genes from pairwise comparison. Three different stages of *G* *. australe* and *G* *. arboreum* seed germination were compared using the pairwise comparison method; up- and downregulated *Unigenes* are indicated.

To further identify genes exhibiting significant differences between the libraries, pairwise comparison was carried out and significance was confirmed using edgeR. In total, we identified 13,884 differentially expressed genes through all three germination stages and between the two cotton species, with an FDR cutoff of 0.001 and |log2Ratio| ≥ 1. A total of 7,146 (51.5%) DEGs were annotated using the older BLASTx procedure, leaving nearly half of the DEGs unannotated; these DEGs were considered to possibly represent novel transcripts, fragments or long noncoding RNAs. We used an FPKM cutoff of 1 to consider a gene to be expressed. The detailed relationships between expressed genes and differentially expressed genes are shown in Figure **5**. A total of 1,945 (1,144 + 801), 339 and 873 *Unigenes* were specifically regulated DEGs corresponding to the three stages of A1, A2 and A3, respectively, while 2,808 (1,918 + 890), 460 and 1,755 *Unigenes* were specifically regulated corresponding to the G1, G2 and G3 stages, respectively, and 4,700 and 3,584 *Unigenes* corresponding to *G* *. arboreum* and *G* *. australe* , respectively, were coexpressed DEGs through all three germination stages. The distributions of up- and downregulated *Unigenes* through nine pairwise comparisons are shown in Figure **6**. The number of DEGs detected in same-stage comparisons between the two cotton species was generally greater than that detected from same-species comparisons at different stages, indicating the huge differences between the two genome types and their regulatory patterns. Pigment glands appear in the third stage of *G* *. australe* seeds germination (G3), while seeds at the first stage (G1) do not contain glands. We also considered the middle stage of the developmental process to help elucidate the dynamic mechanisms of both seed germination and gland formation. The number of DEGs identified in the G1 vs. G3 comparison was remarkably higher than that detected between G1 and G2 and between G2 and G3, providing valuable resources for further elucidating the complicated regulatory mechanisms that occur during germination and gland development.

### Clustering and Functional Enrichment of DEGs

**Figure 7:**
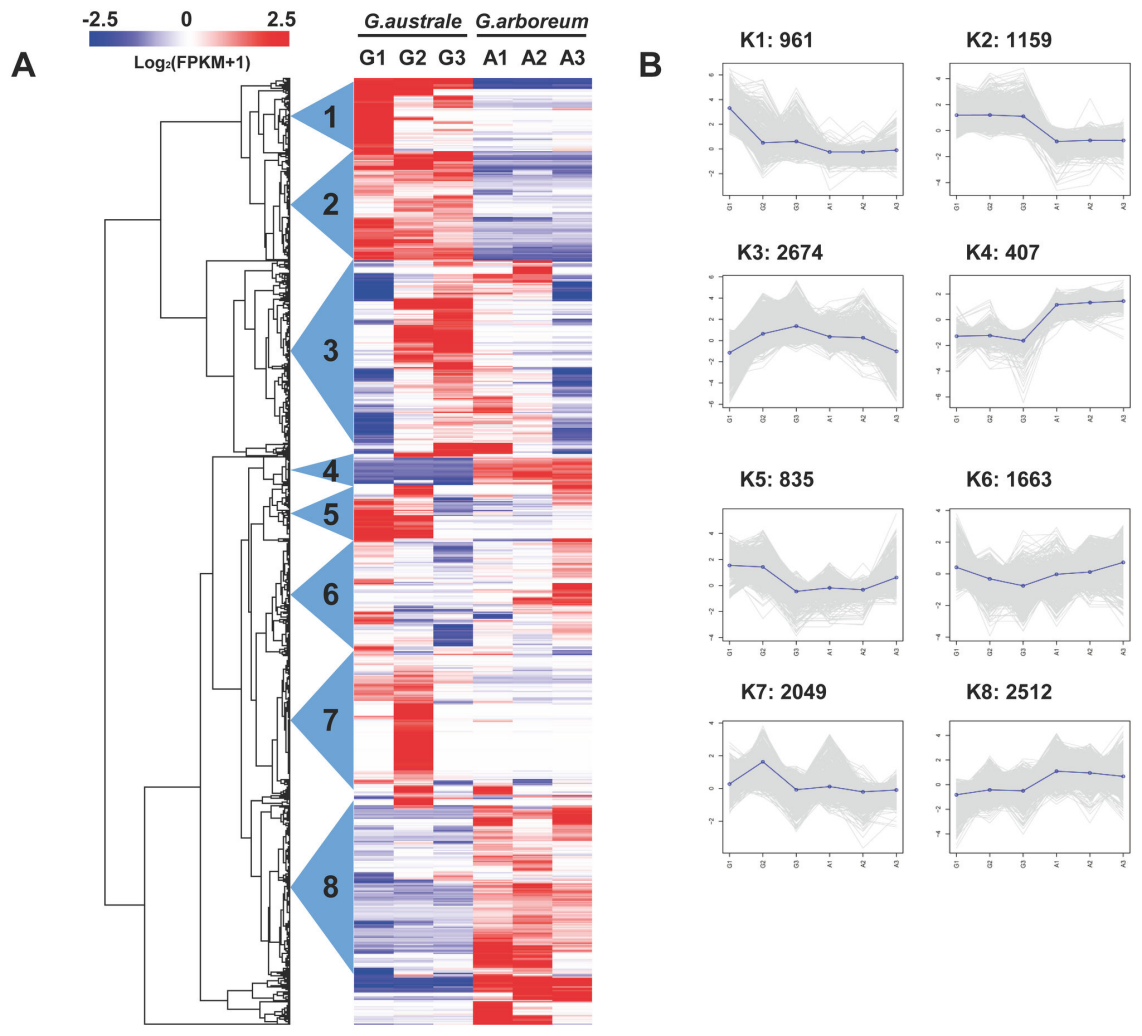
Hierarchical clustering of DEGs. (A) Heatmap plot of DEGs using the hierarchical clustering method; eight main clusters are shown; expression values of six individual germination stages are presented after being centered and log-transformed; decreased (blue) and increased (red) expression of DEGs are distinguished from different species and stages; (B) Expression patterns of genes in the eight main clusters, namely K1-K8, corresponding to the hierarchical heatmap.

**Figure 8:**
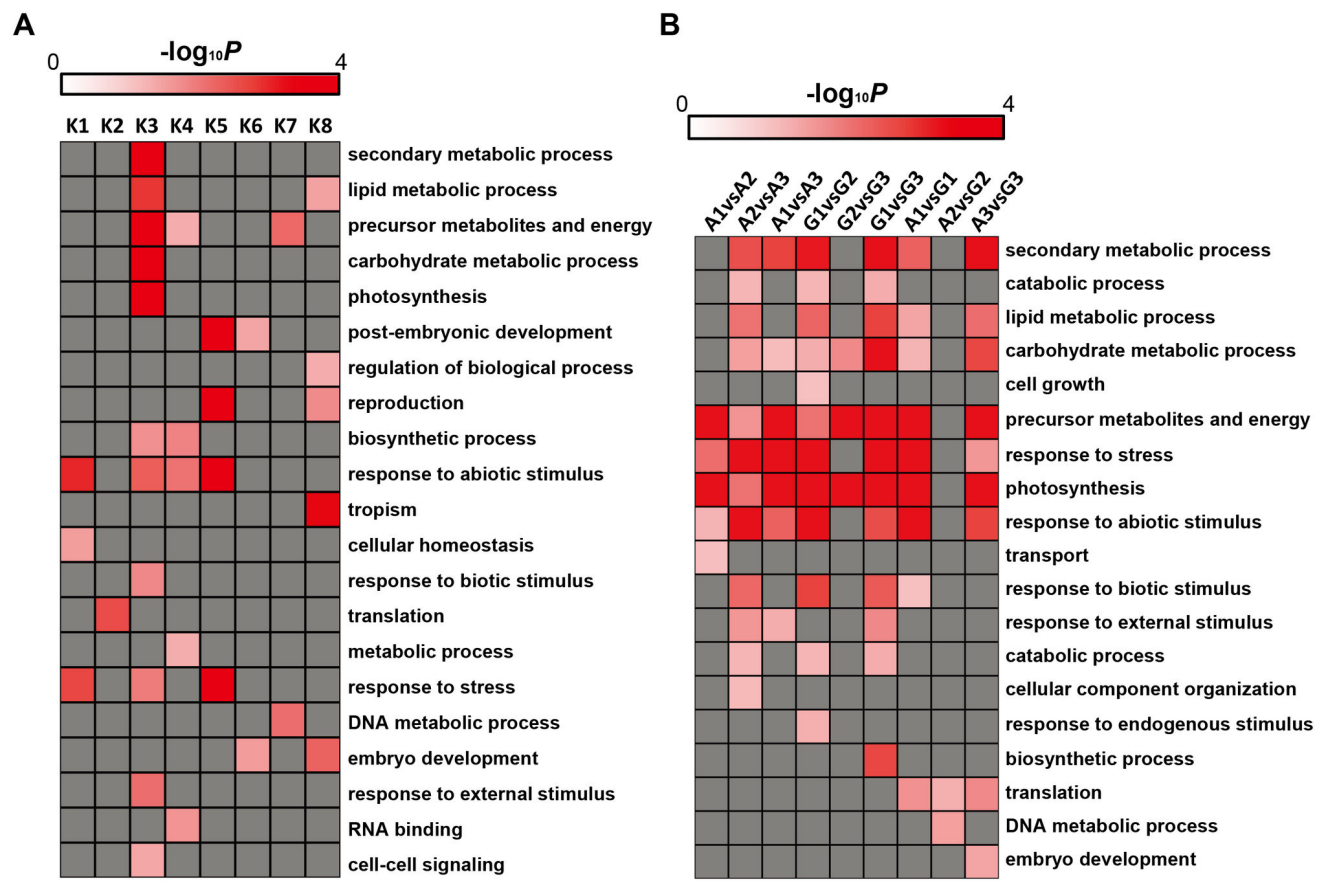
GO-term function enrichment analysis of different clusters, and pairwise comparisons. (A) (B) represent the GO-term enrichment of eight main clusters and nine pairwise comparisons; the significances of the most represented GO-slims in each main cluster and comparison pair are indicated using log-transformed P-value (red), the dark grey areas represented the missing values; (B) GO-term enrichment of nine pairwise comparisons.

We performed hierarchical clustering of the DEGs (Figure **7A**) using the Euclidean distance method associated with complete-linkage, hoping to further illustrate the relationships between DEGs with various expression patterns. We self-defined 16 clusters according to the cluster results, and eight main clusters, accounting for 90% of the DEGs, were plotted with expression patterns (Figure **7B**). The K3 cluster possessed the most genes (2,674); the majority of these genes (821 of 2,674) showed upregulation in *G* *. australe* and downregulation in *G* *. arboreum* . GO-enrichment was performed against all of the annotated *Unigenes* of the combined assembly. The overrepresented GO-slim terms of DEGs in biological process are shown in Table **3**. Many of the DEGs are involved in metabolism process, as well as energy and binding activities. We further analyzed the overrepresented GO functions within each main cluster; the enriched GO terms of biological process are showed in Figure **8A**. The K3 cluster contained the most overrepresented GO terms among all of the clusters. Genes involving secondary metabolic process, lipid metabolic process and generation of precursor metabolites and energy were greatly enriched in this cluster. These results suggest that not only is there a delay in gland development in *G* *. australe* , but the genes that exhibit opposite regulatory patterns in this species may also affect many other traits.

**Table 3:** Overrepresented GO-terms of DEGs.

**GO IDs**	**Function description of biological process**	***P*-value**
**GO:0015979**	Photosynthesis	1.74E-12
**GO:0006091**	Generation of precursor metabolites and energy	3.34E-06
**GO:0009628**	Response to abiotic stimulus	6.20E-06
**GO:0006629**	Lipid metabolic process	4.38E-05
**GO:0008152**	Metabolic process	3.73E-04
**GO:0019748**	Secondary metabolic process	6.87E-04
**GO:0009058**	Biosynthetic process	9.37E-04
**GO:0006950**	Response to stress	1.63E-03
**GO:0005975**	Carbohydrate metabolic process	2.28E-03
**GO:0006412**	Translation	3.45E-03
**GO:0044249**	Cellular biosynthetic process	3.45E-03
**GO:0009059**	Macromolecule biosynthetic process	3.45E-03
**GO:0034645**	Cellular macromolecule biosynthetic process	3.45E-03
**GO:0050896**	Response to stimulus	6.31E-03

Pairwise comparisons between different species and between different germination stages can provide clues about the complexity of seed germination and gland formation. We therefore carried out enrichment analysis of the samples. More genes were enriched in particular GO terms than were revealed in the hierarchical clustering results, indicating the dynamics of seed development. The main GO terms overrepresented in the G1 vs. G3 pair include photosynthesis, secondary metabolic process, generation of precursor metabolites and energy (Figure **8B**). Genes that encode enzymes for secondary metabolism, response to stimulus, lipid metabolism and carbohydrates were greatly enriched in both the cluster analysis and the pairwise comparisons. Taken together, these results reveal the dynamic processes that occur during seed germination and the development of diverse traits.

**Figure 9:**
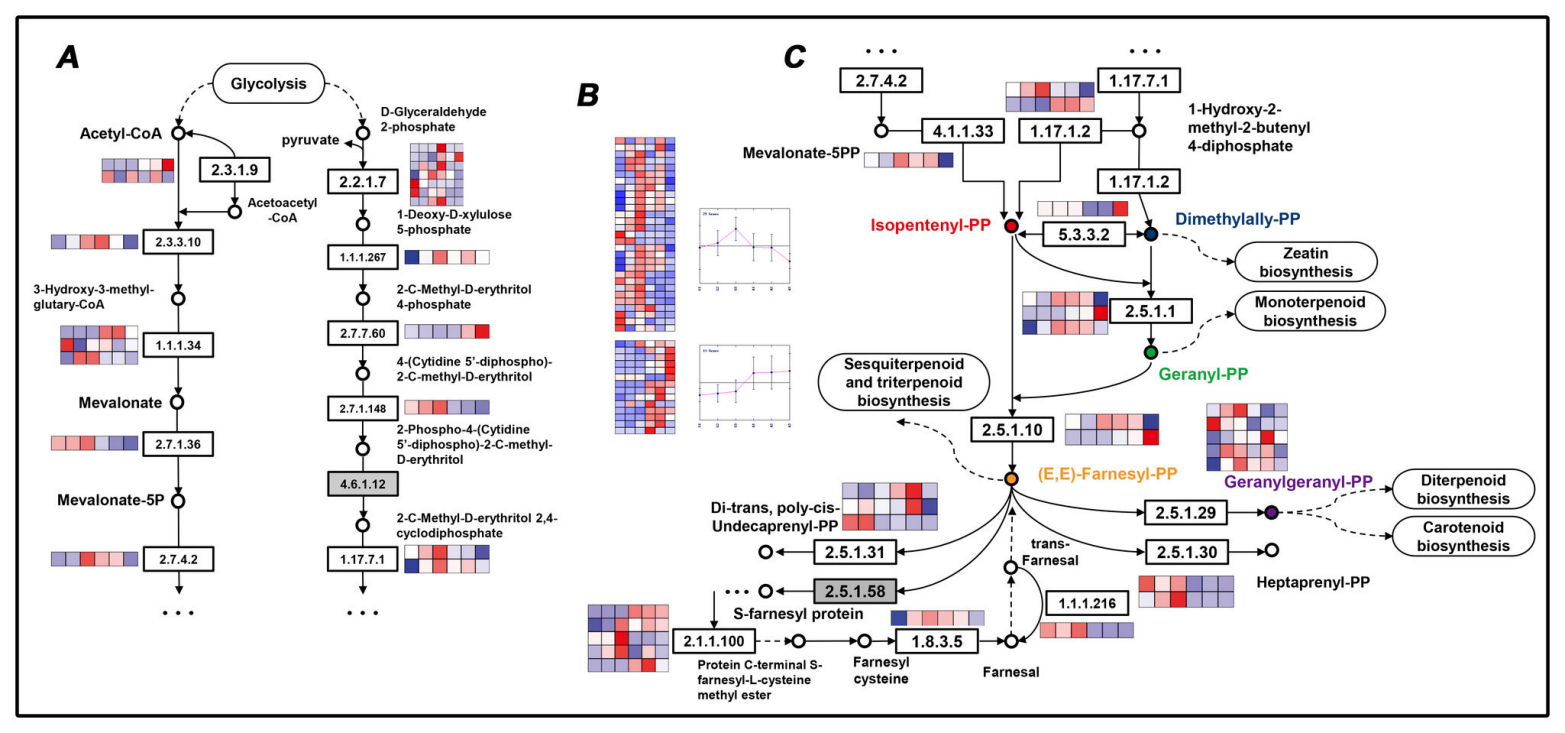
Differentially expressed *Unigenes* assigned to terpenoid backbone biosynthesis. (A) Expression patterns of enzymes within the MEV and MEP/DOXP pathways. (B) Two clusters of expression patterns for all enzymes assigned to terpenoid backbone biosynthesis. (C) Expression patterns of enzymes within downstream biosynthesis pathways (IPP, GPP, FPP, GGPP). The expression heatmaps are arranged in the following order: G1, G2, G3, A1, A2, A3. The log-transformed expression values range from -1 to 1.

To further explore the biological pathways that involve the differentially expressed genes, we performed KEGG analysis of DEGs using the online annotation software “KAAS”. We detected dozens of genes related to biosynthesis of secondary metabolites, especially pathways accounting for terpenoid synthesis and starch and sucrose metabolism processes. Interestingly, when we combined the annotation results of both the whole transcriptome and the DEGs, we found that the sesquiterpenoid biosynthesis pathway, which accounts for the production of most of the gossypol composition [18-22,27], was not particularly enriched, but the expression levels of cadinene synthase genes were relatively high. These results indicate that cadinene synthases is required for the biosynthesis of gossypol. We found that the upstream biosynthesis pathways were relatively active among the DEGs, such as starch and sucrose metabolism (379 *Unigenes* assigned), the glycolysis/gluconeogenesis pathway (118 *Unigenes* assigned) and the mevalonate (MEV) and MEP/DOXP pathways (43 unigene assigned) within terpenoid backbone biosynthesis. Interestingly, we found that a fairly large number of *Unigenes* assigned to the terpenoid backbone biosynthesis pathways showed completely opposite expression patterns between *G* *. australe* and *G* *. arboreum* . Figure **9** indicates the expression levels of genes that encode enzymes in the terpenoid backbone biosynthesis pathways. In addition, we carried out hierarchical clustering of all of these genes. The first cluster is enriched in genes that exhibit opposite expression patterns in *G* *. australe* and *G* *. arboreum* ; the differential expression of these genes may help explain the delayed development of gossypol and glands in *G* *. australe* . The results also suggest that the key genes that regulate gland formation may encode upstream regulatory factors that have a huge impact on downstream pathways. We found that only (E, E)-farnesyl diphosophate synthase (EC:2.5.1.1 2.5.1.10) is responsible for sesquiterpenoid and triterpenoid biosynthesis, which is consistent with the fact that gossypol is the product of cyclization of (E, E)-farnesyl diphosophate to (+)-delta-cadinene, which is later converted to 8-hydroxy-(+)-delta-cadinene [24,74].

### Candidate Transcription Factors for Pigment Gland Formation

Transcription factors play important roles in regulation. Studies have shown that various transcription factors may be important for the formation of gossypol and the development of pigment glands [27-29]. Therefore, in this study, we were interested in describing the distribution and expression patterns of transcription factor genes in both cotton species at various stages. First, we obtained sequence data for transcription factors from 10 plant species (See Materials and Methods) from the PlantTFDB (http://planttfdb.cbi.edu.cn). The BLASTx algorithm was then performed using the assembled transcriptomes. We identified 3,725 (“A”), 2,735 (“G”) and 4,185 (“A & G”) possible transcription factors, 1,253 of which were differentially expressed among samples (Figure **S1A**). The largest category of differentially expressed TF genes encodes MYB/MYB-related transcription factors, followed by NAC.

The *Gl*_*2*_^*e*^ gene has previously been fine mapped between two distinguished SSR markers, NAU2251b and CIR362. To better understand the mechanism of pigment gland formation, we extracted the genome sequences of *G* *. raimondii* between the two markers. We used FGENESH+ gene model prediction software to predict the possible ORFs between the markers; this analysis returned 137 predictions. The predicted ORFs were also assigned to the transcription factor database. We detected 27 transcription factors between the markers, including ERF, MYB, GRF, NAC, Trihelix, zf-HD, bHLH, co-like, WRKY, ARR-b and others. We then used the detected TF sequences to search against the combined assembled *Unigenes* with BLASTn. This analysis returned 23 hit sequences. The regulatory patterns and expression levels of these candidate transcription factors were compared using a hierarchal clustering algorithm (Figure **S1B**).

### Phylogenetic Analysis of Plant Terpene Synthases

Terpenoids, the largest family of secondary metabolic compounds, function in various plant defense and attraction reactions. Previous studies have demonstrated that (+)-delta-cadinene synthase is mainly responsible for the accumulation of sesquiterpenes in cotton. In this study, we further explored the cotton terpene synthase family. The *Getorf* program, available in the EMBOSS software package, was used to extract the predicted ORF sequences in the transcriptome assemblies of *G* *. arboreum* and *G* *. australe* . We used HMMER3.0 to further determine possible terpene synthase gene sequences. The protein sequences predicted in the *G* *. raimondii* genome project were also used to extract full-length terpene synthase-like sequences. Validation of the extracted sequences was carried out by searching against the Non-redundant (NR) databases in NCBI using BLASTp. Moreover, sequences of other plant species such as *Vitis vinifera,* *Populus* *trichocarpa* *,* *Selaginella* *moellendorffii* *,* *Physcomitrella* *patens* *, Arabidopsis thaliana, Oryza sativa* and others were also obtained from NCBI. Terpene synthases genes (TPSs) can be generally divided into seven subfamilies, i.e., TPS-a to TPS-g. Chen et al., defined another TPS-h subfamily specifically for the TPSs identified in *Selaginella* *moellendorffii* . TPS-e/f subfamilies are maimly found in vascular plants. The TPS-d group is the only group that was not detected within the cotton TPS family; this group is only found in gymnosperms [17]. A phylogenetic tree of all aligned TPSs is shown in Figure **S2** and the distribution of TPSs identified in three species i.e. *G* *. raimondii* , *G* *. australe* and *G* *. arboreum* are presented in Table 4. The most dominant subfamily in *G* *. raimondii* is TPS-a, and 44 TPSs were identified in the genome sequences. TPS-a is mainly responsible for the synthesis of sesquiterpenes and is considered to be the most diverse TPSs among the whole family. Seven and five TPSs were identified as TPS-a in *G* *. arboreum* and *G, australe*, respectively. Many TPSs showed tissue specific expression and thus may explain the limited number of TPSs detected in the two species. TPS-b subfamily is mainly responsible for the synthesis of monoterpenes and ranks the second in *Gossypium* TPSs subfamilies. Interestingly, only one TPS-g like sequence can be found in all three cotton species, indicating the importance of this unique TPS gene. TPS-g subfamily is thought to be mainly responsible for the synthesis of monoterpenes and the lack of RRx _8_W motif makes it distinguished from TPS-b subfamily.

**Table 4:** Statistics of TPSs classification in three species.

**TPS subfamilies**	** *G* *. raimondii* **	** *G* *. australe* **	** *G* *. arboreum* **
**TPS-a**	44	5	7
**TPS-b**	25	2	2
**TPS-c**	6	0	1
**TPS-d**	0	0	0
**TPS-e/f**	5	1	1
**TPS-g**	1	1	1

### qRT-PCR Validation

We also used RNA samples isolated for RNA sequencing to perform qRT-PCR analysis. The cadinene synthase genes are mainly responsible for sesquiterpenoid accumulation and have an impact on gossypol synthesis. We therefore employed three of these genes (i.e., comp62156_c1_seq1, comp78894_c1_seq1 and comp73507_c0_seq1), as well as two randomly chosen differentially expressed genes that encode transcription factors, for qPCR validation. The results of qPCR and RNA-seq were consistent. Both experiments showed that the expression patterns of cadinene synthase genes in *G* *. australe* vs. *G* *. arboreum* were quite different (Figure **S3**). We then analyzed the tissue-specific expression patterns of comp78894_c1_seq1 using qRT-PCR. This gene exhibited the highest expression levels among all cadinene synthase genes that were detected and may be the gene that is primarily responsible for the accumulation of gossypol in *G* *. australe* and *G* *. arboreum* . The results show that this unigene was specifically expressed in roots, which provides powerful evidence for the notion that gossypol is synthesized and accumulated in roots and is then transported to the aboveground parts of *Gossypium* [3].

There are two possible explanations for the observation that the seeds of *G* *. australe* lack gossypol. First, certain transportation processes may be blocked in this species, thus preventing the gossypol from getting to the ovules, resulting in the production of gossypol-free seeds. Second, as glands are the storage organs of gossypol, the delayed gland morphogenesis observed in *G* *. australe* may lead to the production of glandless seeds. Therefore, even if gossypol is synthesized in this species, it would not be able to accumulate at the proper destination. More interestingly, the E genome species *Gossypium* *stocksii* exhibits a unique characteristic, namely, dormant seeds of this species are covered with glands but contain no gossypol. This observation suggests that the relationship between glands and gossypol development is quite complex [75]. Zhu et al. examined the anatomical structures of several Australian wild cotton species using scanning electron microscopy. Their results showed that although glands were invisible to the naked eye in these species, special cells comprising lysigenous cavities, referred to as the “gland primordia”, were observed in the glandless, gossypol-free seeds of these species. The disintegration of these cells during germination leads to the appearance of glands [76,77]. Further studies will be needed to further explore the mechanisms underlying both the accumulation and transportation of gossypol and gland formation.

## Conclusions

The presence of glands and gossypol are two related but distinguishable characteristics of cotton. The delayed gland morphogenesis trait in *G* *. australe* makes this plant an ideal model for studying gland and gossypol formation. Our results show that the upstream pathways of terpenoid compound synthesis are delayed in *G* *. australe* , resulting in a delay in gossypol synthesis and gland appearance. We also identified candidate genes that are related to this process. The results provide evidence for key genes that regulate gossypol synthesis and gland formation. Some of the genes encoding upstream regulatory factors that exhibited large differences in expression levels may be responsible for these processes. In addition, the data provide us with powerful resources to further elucidate the biological processes that occur during seed germination, gland formation and gossypol synthesis.

## Supporting Information

Figure S1 **Distribution of differentially expressed transcription factors and expression heatmap of candidate TFs.** (A) Distribution of differentially expressed transcription factors during seed germination. (B) Expression heatmap of candidate transcription factors. The expression heatmaps are arranged in the following order: G1, G2, G3, A1, A2, and A3. The log-transformed expression values range from -1 to 1.
(TIF)

Figure S2 **Phylogenetic analysis and subfamily classification of Terpene Synthase genes** **(TPSs)**. TPSs can be classified into seven main subfamilies, i.e., TPS-a to TPS-g. Terpene synthase genes derived from *G* *. raimondii* , *G* *. arboreum* , *G* *. australe* and other plant terpene synthase genes were used to generate the phylogenetic tree. The bootstrap value was set to 1000.
(TIF)

Figure S3 **Relative expression values of chosen *Unigenes* .** Expression values of all stages were compared to that of A1 for relative comparison purposes; the expression pattern results were consistent between qRT-PCR and RNA-seq analysis. Tissue-specific expression validation of comp78894_c1_seq1 was carried out. The relative expression levels (compared to Roots) of Roots (R), Stems (S), Leaves (L) and 10-DPA Ovules are shown.
(TIF)
