# Supplementary figures and images for: Transcriptome Sequencing and Differential Gene Expression Analysis of Delayed Gland Morphogenesis in Gossypium australe during Seed Germination
Source: PLoS One. 2013 Sep 20;8(9):e75323. doi: 10.1371/journal.pone.0075323 (PMC3779162; doi:10.1371/journal.pone.0075323)

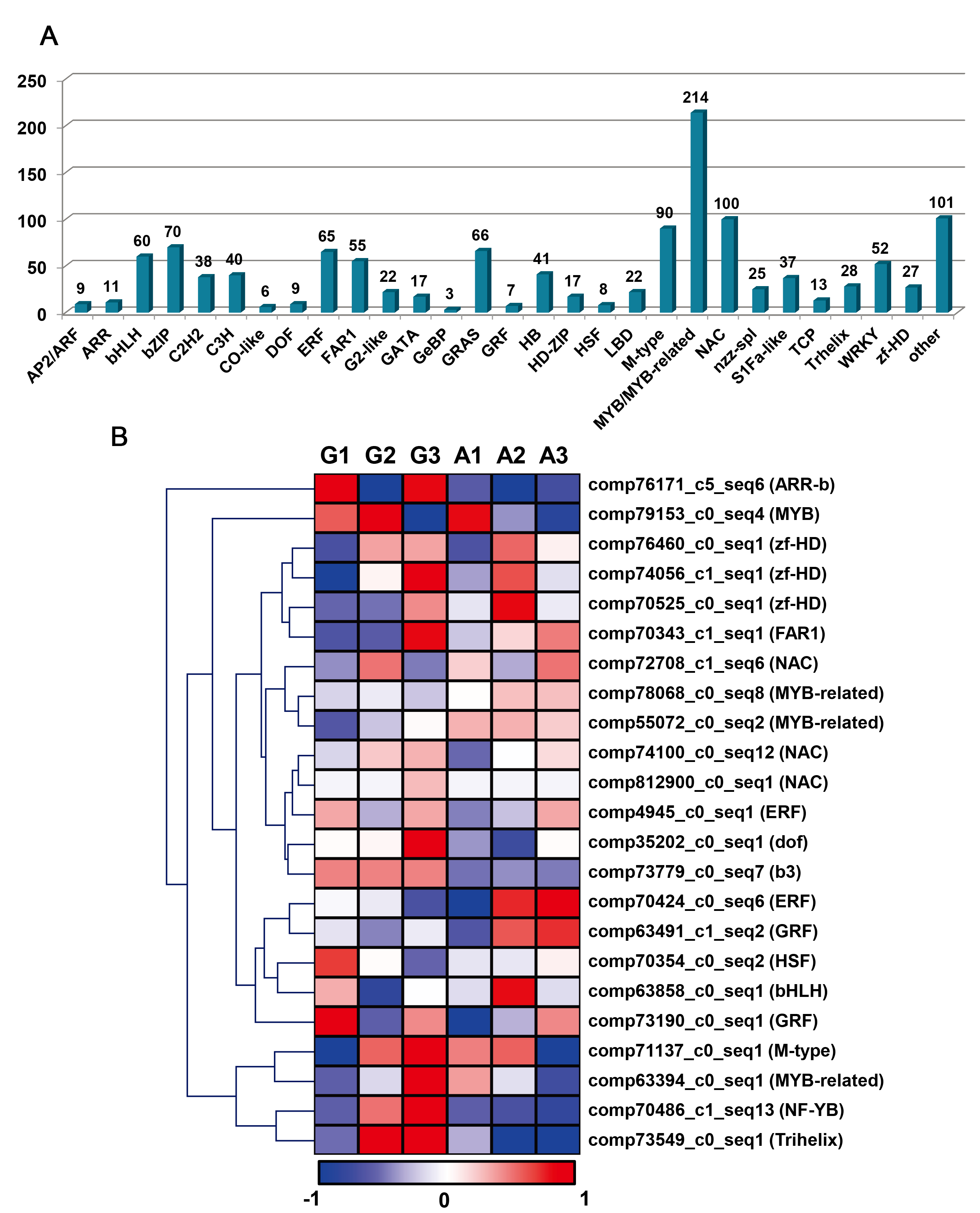

Supplement: Figure S1 — Distribution of differentially expressed transcription factors and expression heatmap of candidate TFs. (A) Distribution of differentially expressed transcription factors during seed germination. (B) Expression heatmap of candidate transcription factors. The expression heatmaps are arranged in the following order: G1, G2, G3, A1, A2, and A3. The log-transformed expression values range from -1 to 1. (TIF) [file pone.0075323.s001.tif]

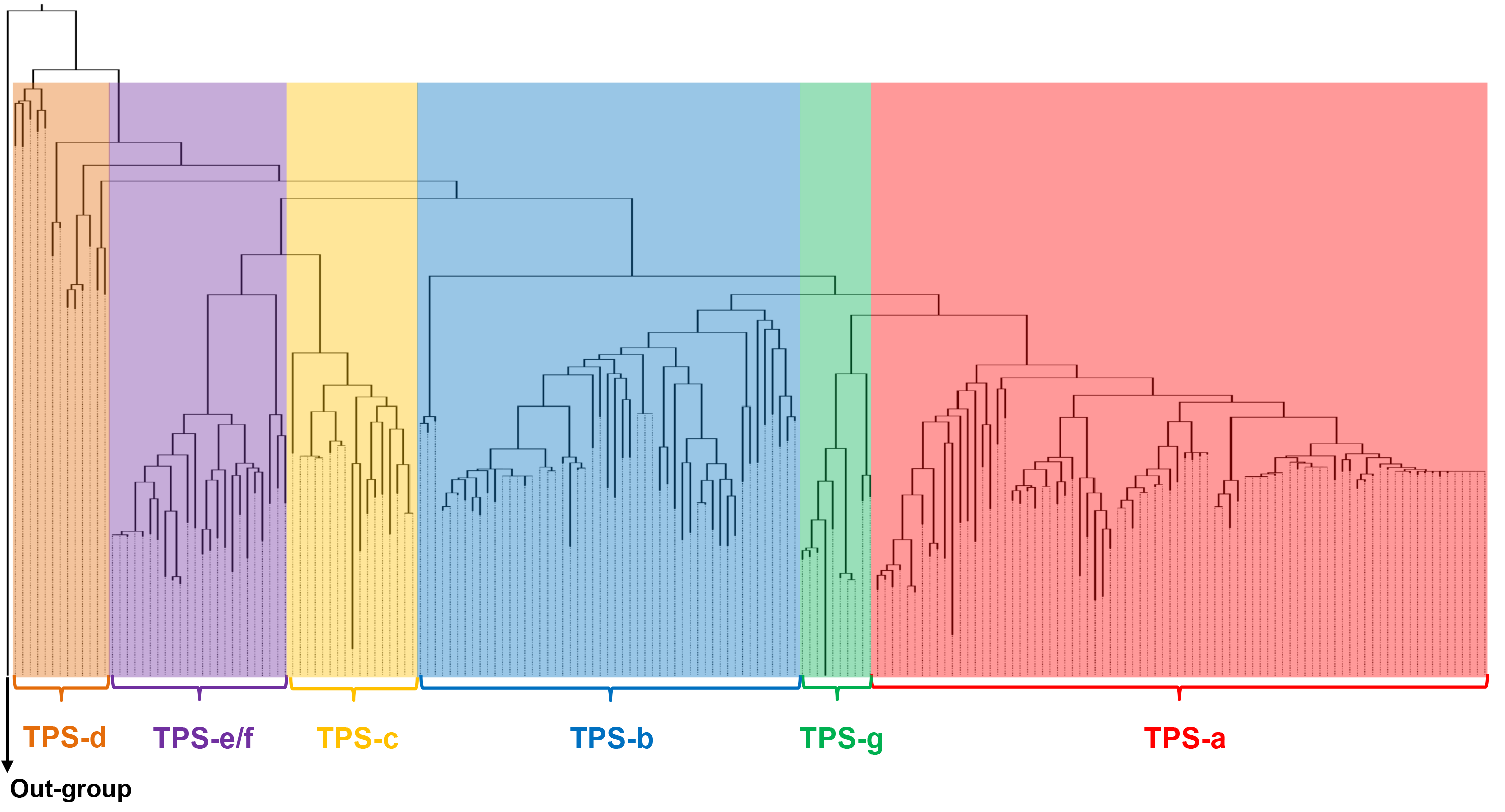

Supplement: Figure S2 — Phylogenetic analysis and subfamily classification of Terpene Synthase genes (TPSs). TPSs can be classified into seven main subfamilies, i.e., TPS-a to TPS-g. Terpene synthase genes derived from G . raimondii , G . arboreum , G . australe and other plant terpene synthase genes were used to generate the phylogenetic tree. The bootstrap value was set to 1000. (TIF) [file pone.0075323.s002.tif]

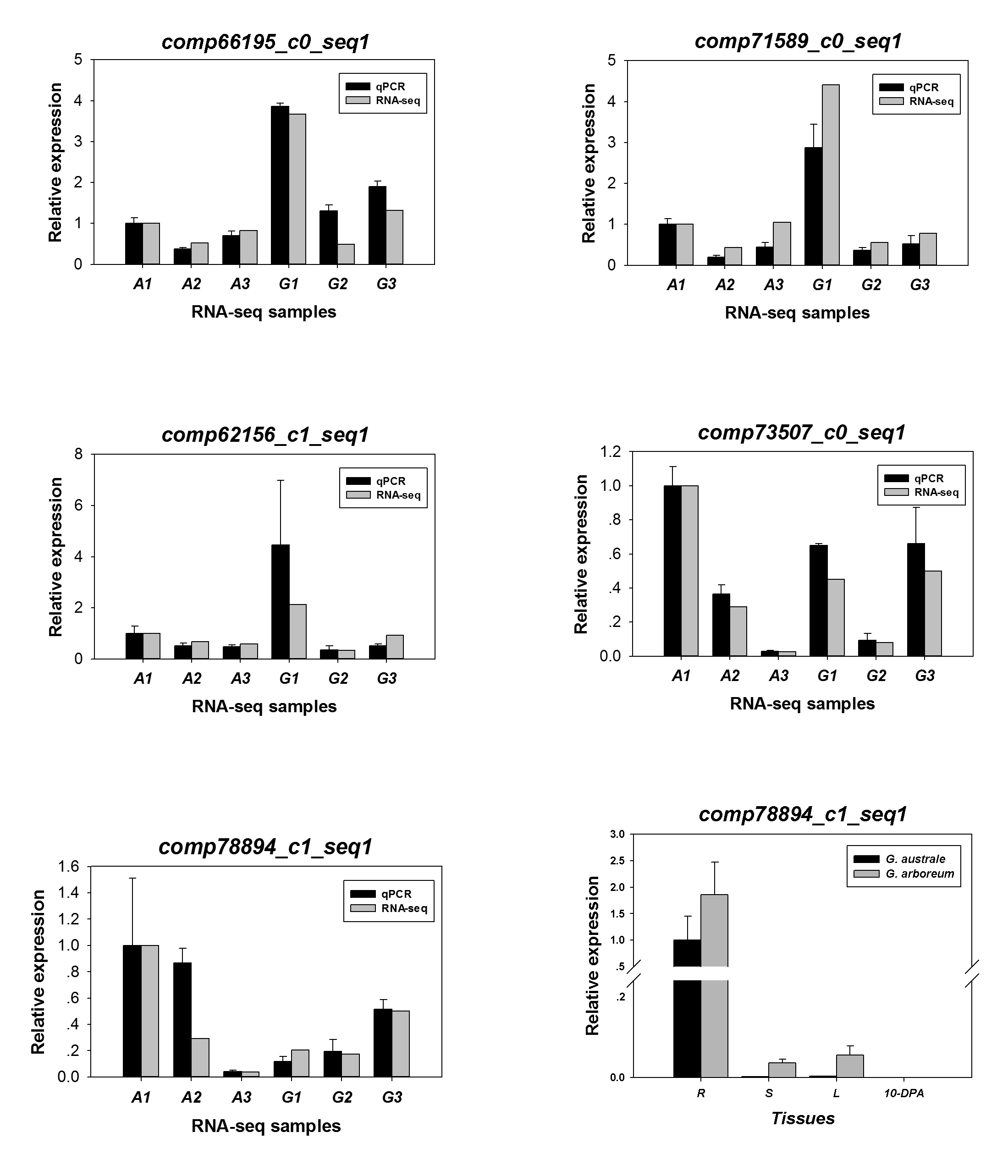

Supplement: Figure S3 — Relative expression values of chosen Unigenes . Expression values of all stages were compared to that of A1 for relative comparison purposes; the expression pattern results were consistent between qRT-PCR and RNA-seq analysis. Tissue-specific expression validation of comp78894_c1_seq1 was carried out. The relative expression levels (compared to Roots) of Roots (R), Stems (S), Leaves (L) and 10-DPA Ovules are shown. (TIF) [file pone.0075323.s003.tif]
